# EXPLORING THE ROLE OF SOCIAL FACTORS AND CHANGES IN RIDE-HAIL (UBER/LYFT) USE AMONG OLDER ADULTS

**DOI:** 10.1093/geroni/igad104.1983

**Published:** 2023-12-21

**Authors:** Jonathon Vivoda, Rosa Grippa

**Affiliations:** Miami University, Oxford, Ohio, United States; Miami University, Oxford, Ohio, United States

## Abstract

Previous research has observed low ride-hail (e.g., Uber) use among older adults and identified some factors related to use. However, uptake of technology changes quickly and pandemic-related factors (e.g., social distancing) may have affected ride-hail use. The Pew Research Center’s American Trends Panel (ATP) datasets include variables related to political and social views, behavior, religion, geography, internet use, and other factors that have not been previously assessed in relation to ride-hailing among older adults. ATP waves from 2015, 2018, and 2021 included questions about ride-hail use, and were assessed in this study. In 2015, 15.4% of all respondents and 4.5% of older adults reported having ever used ride-hailing. In 2018, the numbers had increased to 36.1% overall and 18.1% for older adults. In 2021, participants were only asked about ride-hail use during the past year, with 22.4% overall and 8.7% of older adults reporting ride-hail use. Separate logistic regression models were used to determine factors associated with reporting ever using ride-hail services (2018) and having used ride-hailing during the past year (2021), among older adults. Higher odds of reporting any use (2018) were related to living in a metropolitan area, living in the US’s West, attending religious service monthly vs. never, high income, more liberal ideology, female gender, and volunteering. Higher odds of use during the past year (2021) were related to metropolitan area, male gender, more education, Hispanic ethnicity, divorced and single vs. married, unaffiliated vs Catholic religion, less frequent religious attendance, high income, and frequent internet use.

